# Are Health Literacy and Lifestyle of Undergraduates Related to the Educational Field? An Italian Survey

**DOI:** 10.3390/ijerph17186654

**Published:** 2020-09-12

**Authors:** Francesca Gallè, Patrizia Calella, Christian Napoli, Fabrizio Liguori, Eduardo Alfonso Parisi, Giovanni Battista Orsi, Giorgio Liguori, Giuliana Valerio

**Affiliations:** 1 Department of Movement Sciences and Wellbeing, University of Naples “Parthenope”, 80133 Naples, Italy; patrizia.calella@uniparthenope.it (P.C.); fabrizio.liguori91@gmail.com (F.L.); giorgio.liguori@uniparthenope.it (G.L.); giuliana.valerio@uniparthenope.it (G.V.); 2 Department of Medical Surgical Sciences and Translational Medicine, “Sapienza” University of Rome, 00189 Rome, Italy; christian.napoli@uniroma1.it (C.N.); eduardoparisiroma@gmail.com (E.A.P.); 3 Department of Public Health and Infectious Diseases, “Sapienza” University of Rome, 00185 Rome, Italy; giovanni.orsi@uniroma1.it

**Keywords:** health literacy, undergraduates, lifestyle, Mediterranean diet, physical activity

## Abstract

*Background*: Health literacy (HL) is a fundamental ability to successfully deal with health and illness issues. This study aimed to assess HL among undergraduates from healthcare and non-healthcare degree courses of two Italian universities and the association between their HL, lifestyles, and BMI assumed as health outcome. *Methods*: The Health Literacy Assessment Tool (HLAT-8) and the Newest Vital Sign (NVS) were used to assess health literacy dimensions. Demographic and anthropometric data, adherence to Mediterranean diet (MD), physical activity levels, and smoking habits were assessed in the enrolled sample to highlight possible associations. *Results*: A total sample of 806 undergraduates (46% males, mean age 21.01 ± 1.78 years) was recruited. Higher HL scores were found among healthcare rather than non-healthcare students (28.7 ± 4.5 vs. 26.7 ± 4.2 for HLAT-8 and 4.9 ± 1.5 vs. 3.9 ± 1.8 for NVS, *p* < 0.01). However, healthcare undergraduates were more likely to report unhealthy behaviors. Body Mass Index (BMI) was associated with literacy and numeracy skills only in non-healthcare undergraduates. Significant associations were found between HL scores and adherence to MD in both groups. In the regression analysis, educational field and MD were shown to be predictors of HL scores. *Conclusions*: Attending a healthcare related degree course was associated with higher HL scores but not with healthy behaviors. This issue should be addressed considering the role that healthcare professionals may have in educating patients towards a healthy lifestyle. Adherence to MD seems to be related to higher HL scores.

## 1. Introduction

Health literacy (HL) is defined as the individual’s ability to successfully deal with health and illness issues [1,2]. It is determined by a set of aspects related to knowledge, motivation, and competences fundamental to make judgments and decisions concerning health care, disease prevention, and health promotion [3,4]. According to Nutbeam (2008), HL includes three levels: a functional literacy, which refers to basic reading and writing skills and to the ability of applying these skills to health-related materials; interactive literacy, which implies more advanced cognitive and social skills in order to obtain and elaborate information from different sources; and, finally, critical literacy, which is needed to critically analyze information and use quality information to make informed decisions about health [5]. In line with these definitions, it is assumed that sufficient HL increases the individuals’ ability to access, appraise, and use health-related information adequately, and to make good choices for their own health, while low levels of HL induce the inappropriate access and use of health resources [6,7]. The HL concept is context-specific: in clinical settings, HL might refer to the personal resources patients need to successfully navigate health services, while, in public health, it might be defined as the set of knowledge and skills needed to prevent disease and to promote health in everyday life [5,8].

Poor HL has been associated with several health outcomes and factors that may impair the functioning of health care systems, such as inadequate access to care, reduced adherence to medication, poorer self-rated health status, higher hospitalization and mortality rates, lack of understanding and use of preventive services, greater use of emergency care, inequity, and increase of health-care costs [9,10,11,12,13,14].

Based on these considerations, the assessment of HL seems to be fundamental in order to implement effective public health policies and educational interventions in a population [15,16,17,18,19,20,21,22]. On the basis of the aims pursued, several dimensions of HL can be evaluated: health numeracy represents the skill to use and interpret mathematical calculations for health purposes, such as taking medication as prescribed; health prose literacy is the ability needed to follow written medical instructions; other dimensions concern skills necessary to seek communication and information regarding health [15]. According to the health literacy skills conceptual framework, all these dimensions can influence individuals’ health-related behaviors, such as diet and physical activity (PA), which can subsequently influence health outcomes, such as Body Mass Index (BMI), morbidity and mortality [15,23,24]. Nevertheless, lifestyle and health status may also contribute to learn, unlearn, reinforce or degrade HL skills in a dynamic process [15].

Since lifestyle is established in the earlier years of a person’s lifespan, developing an adequate HL at an early age is fundamental to adopt those behaviors which characterize a healthy lifestyle—mainly adequate diet, sufficient PA, and no smoking, which can positively affect health in adulthood [25]. Indeed, a strong association between HL, high-risk or unhealthy behaviors, and physical health outcomes (e.g., weight status) was found in adolescents, while studies on young adults are still limited [26,27,28]. Some investigations were performed to evaluate HL among university students attending healthcare related courses in order to improve their core HL curriculum in the perspective of their future role in health education [29,30,31]. However, the characterization of HL among undergraduates attending non healthcare-related courses may be also important in order to prevent unhealthy behaviors such as unbalanced diet, inactivity, smoking and alcohol consumption [32,33,34,35]. The HL of graduates must be adequate so that they can guide others in maintaining and improving their health and well-being, and improve the economy of their country; therefore, educational curricula should consider this item [36].

This study aims to: (i) evaluate the levels of HL, in all its dimensions, among undergraduates attending healthcare and non-healthcare related degree courses in two Italian universities; (ii) identify possible group differences; (iii) investigate the relationship between undergraduates’ HL, health-related behaviors and BMI assumed as health outcome.

## 2. Methods

A cross-sectional study was carried out in 2019 among students from “Parthenope” University of Naples and “Sapienza” University of Rome. The investigation was performed anonymously in accordance with the Italian regulation on personal data protection and with the World Medical Association Declaration of Helsinki. Participants provided voluntarily informed written consent to participation. The study was approved by the Ethical Committee of the University of Campania “Luigi Vanvitelli” (approval number 478/2019).

### 2.1. Participants and Settings

Participants were recruited by convenience from healthcare (nursing) and non-healthcare (economics and movement sciences) degree courses. During lessons, a researcher invited the students to participate in the investigation, explaining the aim of the study and guaranteeing the anonymity of data collection and treatment.

### 2.2. Questionnaires

The questionnaires included a general section regarding demographic characteristics such as gender, age, and degree course. In order to calculate the BMI, participants were also asked to self-report their weight and height values. Two previously validated questionnaires were used to investigate diet and PA habits. The first one, proposed by Sofi et al. [37], was employed to explore the adherence to the Mediterranean diet (MD) model. Participants were asked to provide information regarding their usual daily/weekly consumption of eight food groups (fruit, vegetables, legumes, cereals, fish, meat and meat products, dairy products, alcohol, and olive oil). The second one, the International Physical Activity Questionnaire [38], investigated students’ PA levels as vigorous PA (days per week and minutes per day), moderate PA (days per week and minutes per day), walking (days per week and minutes per day), and sedentary lifestyle (average daily time spent sitting). Finally, a question concerning tobacco smoking (non-smoker or quitter/smoker) was posed.

In order to highlight different issues related to specific HL skills requiring targeted educational interventions [26], two validated questionnaires were used to assess all the domains of participants’ HL: the eight-item Health Literacy Assessment Tool (HLAT-8) developed by Abel et al. [39] and the Newest Vital Sign (NVS), introduced by Weiss et al. [40]. The HLAT-8 explores HL for public health purposes and includes items specifically aimed at measuring the ability of accessing, understanding, evaluating and communicating health-related information. It assesses HL through eight Likert-scale items; a score is attributed to each answer, and the sum of the eight scores indicates the HL level of each respondent. The total score range is 0–37, with higher scores indicating higher levels of HL. The NVS is a quick screening instrument widely used to assess HL by using an ice cream nutrition label that the respondent may hold and review; subsequently, six questions regarding that nutrition label are posed. Four or more correct answers indicate adequate HL, while a score ≤3 indicates limited HL. The NVS is a valid tool to measure literacy and numeracy skills; it is short, accurate, and can be employed in settings where time of administration is limited [41].

### 2.3. Statistical Analysis

A descriptive analysis was carried out on demographic characteristics, declared behaviors, and HL levels. Continuous outcomes were reported as mean values ± standard deviation (SD); data regarding gender and smoking habit were reported as number and percentages. The number of participants with a HLAT-8 score ≥ the 75th percentile value, assumed as an indicator of better literacy, and that of the students reporting an NVS score ≥ to 4, corresponding to an adequate literacy, were also calculated for each group.

The comparison between students from healthcare and non-healthcare degrees was performed through the Student’s *t*-test for continuous outcomes and chi-squared test for non-continuous outcomes. The Cohen’s *d* value was calculated to assess the effect size for these comparisons (small 0.10–0.40, medium 0.50–0.70, large ≥0.80). 

Pearson’s and Spearman correlations were performed either to analyze the association between the two questionnaires employed to assess HL or the association between HL levels and the lifestyle behaviors. 

Multiple logistic regression analyses were performed to identify the significant predictors of each HL score. The dependent variables were built by considering if reported HL scores were ≥75th percentile for HLAT-8 or ≥4 for NVS. Age, gender, MD score, and total weekly PA (categorized as lower or equal/higher than median value), educational field, and BMI (classified as normal weight or overweight/obese) were considered as independent variables.

A value of *p* < 0.05 was assumed as the significance level. Data were analyzed with IBM SPSS version 26 for Windows (SPSS, Chicago, IL, USA).

## 3. Results

Overall, 806 out of 3692 (21.8%) total students attending the enrolled degree courses completed the questionnaires: 262 out of 921 (28.4%) nursing students and 544 out of 2771 (19.6%) economics/movement sciences students. The demographic characteristics, the lifestyle outcomes, and the mean HL scores for both the whole sample and the two subgroups are summarized in Table 1.

As for the variables related to lifestyle, the group of students from the healthcare related degree course showed a significantly lower mean BMI than the other participants, whereas the other variables indicated that a low adherence to MD, inactivity, and smoking habits were more common in this group. In particular, nursing undergraduates reported significantly lower levels of moderate-vigorous PA and higher sitting time. As for the HL levels, the mean HLAT-8 and NVS scores as well as the percentages of individuals with better HL scores were significantly higher in nursing students than in other undergraduates. Medium effect size values were obtained only for age and NVS score differences between groups. Considering the significantly different gender composition of the two groups, the HL levels of healthcare and non-healthcare undergraduates were also compared by gender. Significant differences in HL scores from the two educational fields were confirmed in male as well as in female participants (Supplementary Table S1).

The results of the correlation analyses are reported in Table 2. The scores from the two HL questionnaires showed several correlations with individual characteristics or lifestyle components in both the whole sample and the two subgroups. BMI appeared to be positively correlated to NVS score in undergraduates from non-healthcare related degrees. MD adherence was associated with both HL tools in the total sample and in the two subgroups independently of the questionnaire used, with the only exception of HLAT-8 in nursing students. PA resulted in being positively associated with HL only in nursing undergraduates: total PA correlated with HLAT-8 questionnaire scores, while moderate-vigorous PA correlated with NVS results. No correlations were found for age, smoking habit and sitting time.

The results of the logistic regressions performed considering HL scores as outcomes are shown in Table 3. Reporting a higher HL score by either the HLAT-8 questionnaire or the NVS was significantly associated to the attendance of a healthcare related degree course and to a higher MD adherence. No significant associations were found with age, gender, BMI, or PA.

## 4. Discussion

This study evaluated the levels of HL among Italian undergraduates, comparing results between healthcare and non-healthcare students. We also examined the relationship between HL, lifestyle, and BMI. Our findings show that attending a healthcare related degree course was associated with higher HL levels. The adherence to the Mediterranean diet model seems to be also related to better HL skills.

As indicated by NVS score, 68.8% of the whole sample showed adequate literacy and numeracy, while the communication and information skills evaluated through the HLAT-8 questionnaire were above average, with significantly higher levels found in nursing students. Since its definition, several studies were carried out worldwide to estimate the HL levels of different populations and in different settings [16,17,18,19,20,21,22]. In the United States, the 2003 National Assessment of Adult Literacy reported a 36% rate for low HL in a sample of 14,592 respondent adults [18], while more than 43% of American adults have been reported to have insufficient HL skills [16]. In Europe, a research project entitled “European Health Literacy Survey” (HLS-EU) launched in 2011 to investigate HL in eight European countries through a 86-item survey, showed limited HL skills in about 47% of respondents, with consistent differences (29–62%) across countries [19,22]. As for Italy, recent investigations have reported inadequate HL as a predominant phenomenon; Palumbo et al. investigated HL in a representative sample of 1000 Italian individuals with the same tool used for the HLS-EU [21]. The survey showed an insufficient level of HL in the 54.6% of the sample. A recent study by Lorini et al. carried out on a population-based sample of 223 adults by using three measurement tools reported values of inadequate HL ranging from 40.4% to 75.4% on the basis of the different questionnaire used [20]. In particular, 59.6% of individuals showed an adequate literacy as resulting by the NVS. Therefore, our results suggest that undergraduates, especially those attending healthcare related degree courses, may have a better level of HL respect to the general population. This should be confirmed in future investigations. In 2019, Rüegg and Abel showed that HL may be associated with many material, psychosocial, and health-related factors, and with socioeconomic status. Among the covariates they considered, interest in health topics was included among the best predictors for a good HL level [42]. This finding is in accordance with the results we found among nursing undergraduates.

Furthermore, we analyzed the association between HL, lifestyle, and BMI.

A positive association was found between MD and all the HL dimensions, and the logistic regression suggests that MD may influence HL. The protective role of MD on multiple health outcomes has been widely demonstrated [43]. Moreover, the adoption of this pattern is associated with other healthy habits such as having an active lifestyle and not smoking [44]. In our study, the adherence to a MD pattern was not associated with other lifestyle behaviors (data not shown), but it was related to HL.

According to the study by Geboers et al. [23], we did not find association between HL and smoking. Contrary to the findings of other studies, the HL of our total sample was not clearly related to PA, nor to BMI as physical health outcome [23,26]. In the group analysis, PA was found to be related with HL domains in healthcare students, and BMI was associated with literacy and numeracy skills only in non-healthcare undergraduates. These results highlight the differences existing between the two groups examined and deserve further investigations.

Interestingly, HL and healthcare educational field were found to be associated even if healthcare students showed a lower adherence to healthy lifestyle than their counterparts. Although a direct relation between individuals’ HL skills and health behaviors and outcomes has been demonstrated, literature shows that many factors including health status, attitudes, motivation, and self-efficacy may mediate this relation. Moreover, these mediators may also be influenced by socio-cultural features, support, and available resources [15]. Our results suggest the need to further explore these items in future studies.

The two questionnaires used in this study were used successfully in previous investigations to assess HL among young adults [36,39]. In our experience, they were well accepted and understood by participants, and their results showed a good level of concordance.

### Limitations

This study has some limitations. First of all, the limited size and the composition of the sample do not allow to generalize our findings. The degree courses were chosen by convenience and students participated voluntarily. Therefore, a selection bias should not be excluded. Randomized studies involving more representative samples would confirm our findings. Furthermore, our sample showed a significant difference in gender composition between groups, with females mainly represented among healthcare undergraduates. Although this is an aspect frequently reported in studies carried on the same target population [33,45], differences in HL levels between the two educational fields were maintained when males and females were examined separately, and gender was not significantly associated with HL outcomes in multiple regression analyses. Second, all the data collected were self-reported. It is possible that, besides inaccurate information regarding weight and height, participants reported incorrect estimates of their own HL abilities. Furthermore, we did not analyze the working condition of participants. Considering that being a working student may have effects on PA levels, it should have been investigated as a possible lifestyle predictor [33]. However, the use of two investigation tools and the involvement of undergraduates from two different areas of study represent new items that may contribute to characterize the domains and the determinants of HL. Furthermore, the analysis of the relationships between students’ behaviors and HL opens new perspectives for future research.

## 5. Conclusions

The findings of this study show an adequate HL level in undergraduates, mainly among those attending healthcare-related degree courses. However, the majority of these students did not report healthy behaviors. This issue should be addressed considering the role that healthcare professionals may have in educating patients towards a healthy lifestyle.

Furthermore, in this study, HL was associated with the adherence to the MD pattern. This suggests that having better knowledge and skills regarding health may lead to better food habits. Further investigations regarding these aspects are needed.

## Figures and Tables

**Table 1:** Demographic characteristics and lifestyle of the study participants in the total sample and grouped for areas of study with related *p* and *d* values.

	Total Sample *n* = 806	Health-Related Degree *n* = 262	Non Health-Related Degree *n* = 544	*p*-Value	*d*-Value
Gender *n* (%) femalemale	435 (54) 371 (46)	191 (73) 71 (27)	245 (45) 299 (55)	0.000	0.554
Mean age ± SD Years	21.01 ± 1.78	21.03 ± 2.02	21.01 ± 1.61	0.831	0.010
Mean BMI ± SD kg/m^2^	22.74 ± 3.13	21.95 ± 2.99	23.11 ± 3.13	0.000	0.376
Mean MD adherence score ± SD	8.1 ± 2.9	7.9 ± 3.1	8.1 ± 2.8	0.291	0.069
Smokers *n* (%)	240 (29.7)	80 (30.5)	160 (29.4)	0.332	0.114
Mean total PA time ± SD (min/week)	837±2,292	643±775	930±2,730	0.098	0.126
Moderate-vigorous PA time ± SD (min/week)	352 ± 625	239 ± 329	407 ± 720	0.000	0.271
Walking time ± SD (min/week)	484 ± 2148	402 ± 614	523 ± 2576	0.456	0.056
Sitting time ± SD (min/day)	268 ± 267	332 ± 225	238 ± 280	0.000	0.357
HL score ± SD (HLAT-8)	27.4 ± 4.4	28.7 ± 4.5	26.7 ± 4.2	0.000	0.465
Mean HL score ± SD (NVS)	4.2 ± 1.8	4.9 ± 1.5	3.9 ± 1.8	0.000	0.585
Subjects with HLAT-8 score ≥ 75th percentile *n* (%)	178 (22.0)	92 (35.1)	86 (15.8)	0.000	0.386
Subjects with adequate literacy (NVS) *n* (%)	556 (68.8)	215 (82.1)	341 (62.8)	0.000	0.447

n: number of participants; SD: Standard Deviation; BMI: Body Mass Index; MD: Mediterranean Diet; PA: Physical Activity; HL: Health Literacy; HLAT-8: Health Literacy Assessment Tool; NVS: Newest Vital Sign.

**Table 2:** Results of correlations between HL scores and students’ information in the total sample and in the two subgroups.

	Total Sample *n* = 806		Health-Related Degree *n* = 262		Non Health-Related Degree *n* = 544	
	HLAT-8 Score	NVS Score	HLAT-8 Score	NVS Score	HLAT-8 Score	NVS Score
HLAT-8 score	-	0.191 **	-	0.240 **	-	0.103 *
Age	0.034	0.026	0.069	0.064	0.012	0.053
BMI	−0.035	−0.013	0.020	−0.010	−0.011	0.121 **
MD adherence	0.125 **	0.154 **	0.101	0.159 *	0.156 **	0.178 **
Smoking ^a^	0.051	0.039	0.060	0.069	0.042	0.016
Total PA time	0.034	−0.015	0.123 *	0.087	0.042	−0.009
Moderate-vigorous PA time	0.015	−0.024	0.110	0.123 *	0.030	−0.008
Walking time	0.032	−0.009	0.095	0.046	0.036	−0.007
Sitting time	0.031	0.050	−0.048	0.056	0.006	−0.012

HLAT-8: Health Literacy Assessment Tool; NVS: Newest Vital Sign; BMI: Body Mass Index; MD: Mediterranean Diet; PA: Physical Activity. ^a^ Spearman correlation; *p < 0.05; **p < 0.001.

**Table 3:** Results of the multiple logistic regression performed on Abel and NVS HL scores as outcomes.

	Dependent Variables	
Independent Variables	HL Score (HLAT-8) ≥75th Percentile	HL Score (NVS) ≥4
	OR (95%CI)	
Age	1.056 (0.743–1.501)	1.118 (0.816–1.532)
Gender	0.863 (0.594–1.255)	1.093 (0.787–1.519)
Educational field	3.059 (2.120–4.413) **	2.769 (1.894–4.047) **
BMI	1.007 (0.645–1.572)	1.100 (0.743–1.627)
MD adherence	1.824 (1.289–2.582) **	1.433 (1.042–1.971) *
Total PA time	1.029 (0.676–1.567)	1.438 (0.987–2.094)

HL: Health Literacy; HLAT-8: Health Literacy Assessment Tool; NVS: Newest Vital Sign; BMI: Body Mass Index; MD: Mediterranean Diet; PA: Physical Activity; OR: Odds Ratio; CI: Confidence Interval *p < 0.05; **p < 0.001.

## Supplementary Materials

The following are available online at https://www.mdpi.com/1660-4601/17/18/6654/s1, Table S1: Differences in HL scores of female and male participants grouped by educational field with related *p* and *d* values.
