# Supplementary material for: Are Health Literacy and Lifestyle of Undergraduates Related to the Educational Field? An Italian Survey
Source: Int J Environ Res Public Health. 2020 Sep 12;17(18):6654. doi: 10.3390/ijerph17186654 (PMC7558704; doi:10.3390/ijerph17186654)
Supplement: Supplementary file 1 [file ijerph-17-06654-s001.pdf]

International Journal of
*Environmental Research and Public Health*

MDPI

**Supplementary Table S1.** Differences in HL scores of female and male participants grouped by educational field with related *p* and *d* values.

| | Females | | | | Males | | | |
|---|---|---|---|---|---|---|---|---|
| | **Health-Related Degree** *n* = 191 | **Non Health-Related Degree** *n* = 244 | ***p* Value** | ***d* Value** | **Health-Related Degree** *n* = 71 | **Non Health-Related Degree** *n* = 300 | ***p* Value** | ***d* Value** |
| HL score ± SD (HLAT-8) | 28.5 ± 4.2 | 26.8 ± 4.1 | 0.000 | 0.410 | 29.0 ± 5.2 | 26.7 ± 4.3 | 0.000 | 0.513 |
| Mean HL score ± SD (NVS) | 4.9 ± 1.4 | 3.8 ± 1.8 | 0.000 | 0.672 | 4.9 ± 1.7 | 3.8 ± 1.8 | 0.000 | 0.617 |
| Subjects with HLAT-8 score ≥ 75$^{th}$ percentile *n* (%) | 61 (31.9) | 41 (16.8) | 0.000 | 0.357 | 45 (63.4) | 31 (10.3) | 0.000 | 0.591 |
| Subjects with adequate literacy (NVS) *n* (%) | 160 (83.8) | 152 (62.3) | 0.000 | 0.459 | 55 (77.5) | 189 (63) | 0.017 | 0.241 |
